# Bortezomib in late antibody-mediated kidney transplant rejection (BORTEJECT Study): study protocol for a randomized controlled trial

**DOI:** 10.1186/1745-6215-15-107

**Published:** 2014-04-03

**Authors:** Farsad Eskandary, Gregor Bond, Elisabeth Schwaiger, Zeljko Kikic, Christine Winzer, Markus Wahrmann, Lena Marinova, Helmuth Haslacher, Heinz Regele, Rainer Oberbauer, Georg A Böhmig

**Affiliations:** 1 Division of Nephrology and Dialysis, Department of Medicine III, Medical University Vienna, Währinger Gürtel 18-20, A-1090 Vienna, Austria; 2 Department of Laboratory Medicine, Medical University Vienna, Währinger Gürtel 18-20, 1090 Vienna, Austria; 3 Department of Clinical Pathology, Medical University Vienna, Währinger Gürtel 18-20, 1090 Vienna, Austria; 4 Department of Nephrology, Krankenhaus der Elisabethinen, Fadingerstraße 1, 4020 Linz, Austria

**Keywords:** Antibody-mediated rejection, Donor-specific antibody, Bortezomib, Kidney transplantation, Proteasome inhibition

## Abstract

**Background:**

Despite major advances in transplant medicine, improvements in long-term kidney allograft survival have not been commensurate with those observed shortly after transplantation. The formation of donor-specific antibodies (DSA) and ongoing antibody-mediated rejection (AMR) processes may critically contribute to late graft loss. However, appropriate treatment for late AMR has not yet been defined. There is accumulating evidence that the proteasome inhibitor bortezomib may substantially affect the function and integrity of alloantibody-secreting plasma cells. The impact of this agent on the course of late AMR has not so far been systematically investigated.

**Methods/design:**

The BORTEJECT Study is a randomized controlled trial designed to clarify the impact of intravenous bortezomib on the course of late AMR. In this single-center study (nephrological outpatient service, Medical University Vienna) we plan an initial cross-sectional DSA screening of 1,000 kidney transplant recipients (functioning graft at ≥180 days; estimated glomerular filtration rate (eGFR) >20 ml/minute/1.73 m^2^). DSA-positive recipients will be subjected to kidney allograft biopsy to detect morphological features consistent with AMR. Forty-four patients with biopsy-proven AMR will then be included in a double-blind placebo-controlled intervention trial (1:1 randomization stratified for eGFR and the presence of T-cell-mediated rejection). Patients in the active group will receive two cycles of bortezomib (4 × 1.3 mg/m^2^ over 2 weeks; 3-month interval between cycles). The primary end point will be the course of eGFR over 24 months (intention-to-treat analysis). The sample size was calculated according to the assumption of a 5 ml/minute/1.73 m^2^ difference in eGFR slope (per year) between the two groups (alpha: 0.05; power: 0.8). Secondary endpoints will be DSA levels, protein excretion, measured glomerular filtration rate, transplant and patient survival, and the development of acute and chronic morphological lesions in 24-month protocol biopsies.

**Discussion:**

The impact of anti-humoral treatment on the course of late AMR has not yet been systematically investigated. Based on the hypothesis that proteasome inhibition improves the outcome of DSA-positive late AMR, we suggest that our trial has the potential to provide solid evidence towards the treatment of this type of rejection.

**Trial registration:**

Clinicaltrials.gov: NCT01873157.

## Background

Despite major advances in transplant medicine, which include continuous refinements of immunosuppressive strategies, large registry analyses have failed to demonstrate major improvements in long-term survival of standard kidney transplants over the last decades [1,2]. Recent studies have underscored a dominant role of alloimmune injury as a leading cause of long-term graft loss. In this respect, the formation of antibodies against polymorphic donor antigens, commonly human leukocyte antigens (HLA), has proved to be an important trigger of graft rejection [3-5]. Humoral rejection (antibody-mediated rejection (AMR)) of organ transplants has been established to constitute a separate rejection entity, and in recent years accurate biopsy-based and serological criteria for this rejection type have been defined to provide a solid basis for targeted treatment: microcirculation inflammation and injury; antibody-triggered C4 complement split product deposition (C4d) along peritubular capillaries; and detection of circulating donor-specific antibodies [6,7]. It has become evident that a considerable proportion of recipients develop features of AMR late after transplantation, a process culminating in chronic irreversible tissue damage, graft dysfunction and loss [8-10]. Indeed, there are studies suggesting that newly formed donor-specific antibodies (DSA) represent the primary cause of late graft loss [11-15].

Treatment of AMR is a big challenge. For early acute AMR, various treatment protocols – which include antibody depletion by apheresis, modulation of B-cell immunity by intravenous immunoglobulin (IVIG), or targeting critical components of innate immunity including complement activation – were shown to potentially prevent and reverse rejection [5,16-18].

For late AMR, however, appropriate treatment still remains to be established. A few anecdotal reports and small case series have suggested efficacy of distinct anti-humoral treatment modalities. While uncontrolled studies have suggested stabilization of chronic AMR following treatment with high-dose IVIG and CD20 antibody rituximab, at least in some patients [19-22], other reports have shown that such treatment may not be sufficient to prevent the development of AMR and subsequent chronic injury [23]. A major drawback of currently available treatment strategies may be that they do not directly affect the integrity and function of long-lived alloantibody-producing plasma cells [24].

One attractive treatment concept could be the targeting of alloantibody-producing plasma cells. In this context, the use of bortezomib, a proteasome inhibitor approved for the treatment of multiple myeloma, may be a promising option. There is now increasing evidence that proteasome inhibition could affect nonmalignant autoantibody or alloantibody-secreting cells [25-27]. In recent years, numerous case series and anecdotal reports have suggested efficacy of bortezomib treatment (commonly part of multimodal treatment strategies) in reducing levels of DSA, improving kidney function and preventing graft loss in patients experiencing acute AMR [28-30]. However, there are only scarce data on the efficacy of bortezomib in treating late AMR processes. There are recent experimental and preliminary clinical data suggesting a potential impact of bortezomib also on the course of late AMR [30-33]. In a recently published case of late chronic active AMR with a slow progressive deterioration of kidney function and increasing proteinuria, our study group could also demonstrate a profound downregulation of DSA and a complete abrogation of biopsy-proven antibody-triggered intragraft complement activation following a single cycle of bortezomib [32]. Remarkably, bortezomib treatment was associated with a consistent decrease in proteinuria and stabilization of graft function. As expected, advanced chronic lesions (severe transplant glomerulopathy) in this patient remained unchanged [32]. Such promising results as well a recent experimental model [31] provided a valuable basis for the design of a systematic intervention trial.

## Methods/design

### Trial design

The BORTEJECT Study is an investigator-driven randomized, placebo-controlled single-center trial (phase II) designed to examine the efficiency of the proteasome inhibitor bortezomib in the treatment of late AMR. We hypothesize that, by inhibiting alloantibody production, bortezomib is able to halt the progression of ongoing graft injury and dysfunction caused by antibody-triggered rejection processes. The study will be performed in two major steps. The first step (Part A) consists of a cross-sectional screening analysis of a large cohort of kidney transplant recipients for the presence of late AMR. In a second step (Part B), 44 rejecting recipients will be randomized in a controlled intervention trial. The proposed duration of the trial is 36 months.

### Part A

**Figure 1:**
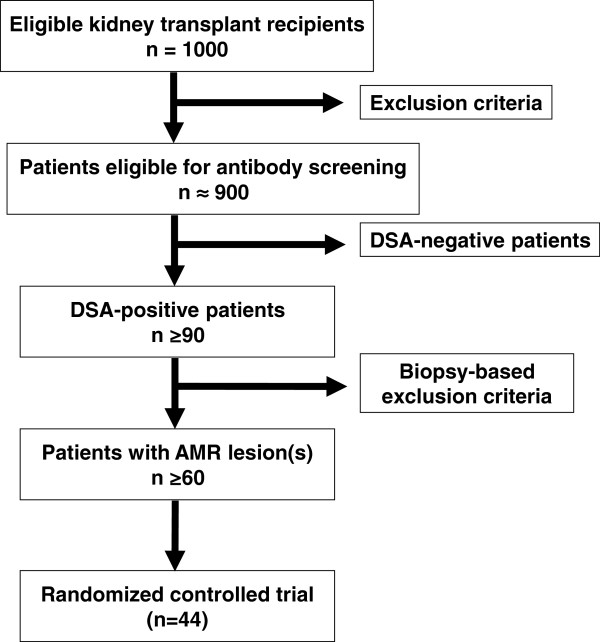
**Study flowchart of Part A (cross-sectional screening).** All kidney transplant recipients from our outpatient service with a functioning allograft at 6 months and estimated glomerular filtration rate >20 ml/minute/1.73 m^2^ will be considered for study inclusion. AMR, antibody-mediated rejection; DSA, donor-specific antibodies.

We plan a cross-sectional screening of approximately 1,000 kidney transplant recipients for the presence of circulating DSA and morphological features of AMR. All patients will be recruited at the outpatient service of the Division of Nephrology and Dialysis, Medical University Vienna. Inclusion and exclusion criteria for this first part of the study are listed in Table 1. Key inclusion criteria are a functioning graft at ≥180 days post transplantation and estimated glomerular filtration rate (eGFR) >20 ml/minute/1.73 m^2^. The glomerular filtration rate (GFR) threshold was chosen to avoid inclusion of transplants with an extensive degree of irreversible chronic damage. We anticipate that approximately 90% of screened recipients will be eligible for serological screening. In at least 10% (*n* ≥ 90) of the HLA antibody-tested patients a positive DSA result can be expected [13]. We estimated biopsy-based AMR features (see Table 2) to occur in at least 60% of the DSA-positive subjects [34]. Hence, our screening can be expected to identify approximately 50 patients eligible for inclusion in the interventional trial (Part B). A flowchart of study Part A is provided in Figure 1.

**Table 1:** Main inclusion and exclusion criteria – Part A

Inclusion criteria	1. Estimated glomerular filtration rate >20 ml/minute/1.73 m^2^
	2. ≥180 days post transplantation
	3. Age >18 years
	4. Written informed consent
Exclusion criteria	1. Acute rejection <1 month before screening
	2. Acute deterioration of graft function suspicious of acute rejection
	3. Documented intolerance of bortezomib, boron or mannitol
	4. Active viral, bacterial or fungal infection
	5. Active malignant disease
	6. Women who are pregnant or breastfeeding
	7. Serious medical or psychiatric illness
	8. Patients actively participating in another clinical trial

**Table 2:** Morphological and immunohistochemical features of AMR

	**AMR features (Banff criteria)**	**Positive result**
1. Histomorphology	Glomerulitis (g)	Banff score^a^ ≥1
	Peritubular capillaritis (ptc)	Banff score^a^ ≥1
	Transplant glomerulopathy (cg)	Banff score^a^ ≥1
2. Immunohistochemistry	Focal/diffuse C4d staining in PTC (C4d)	Banff score^a^ ≥1
3. Electron microscopy	Basement membrane lamellation in PTC	>3 BM layers per PTC

AMR, antibody-mediated rejection; BM, basement membrane; C4d, C4 complement split product deposition; PTC, peritubular capillaries.

^a^Lesions were scored according to the Banff classification of renal pathology [6,7].

### Part B

**Figure 2:**
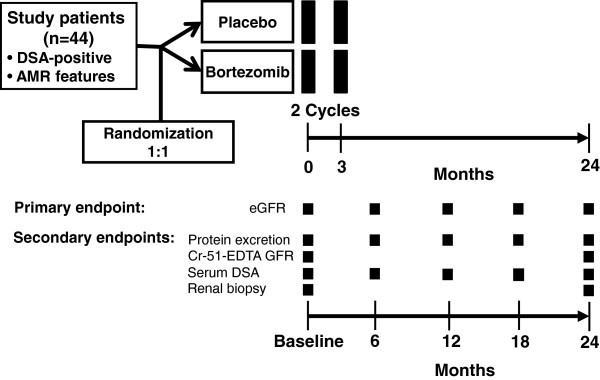
**Study flowchart of Part B (randomized controlled trial).** Forty-four transplant recipients with late biopsy-proven antibody-mediated rejection (AMR) will be randomized to receive either bortezomib or placebo. The primary endpoint, the estimated glomerular filtration rate (eGFR), will be evaluated at 0, 6, 12, 18 and 24 months. Major secondary endpoints are the measured glomerular filtration rate (GFR), protein excretion, patterns of human leukocyte antigen reactivity and results obtained with 24-month protocol biopsies. Cr-EDTA, chromium ethylenediamine tetraacetic acid; DSA, donor-specific antibodies; MFI, mean fluorescence intensity.

The second part of the study is a randomized controlled interventional trial designed to include 44 transplant recipients with late AMR. A flowchart is shown in Figure 2. Inclusion and exclusion criteria are provided in Table 3. Key inclusion criteria are the detection of HLA class I and/or class II DSA and the presence of one or more morphological and/or immunohistochemical features of AMR in the index biopsy.

**Table 3:** Main inclusion and exclusion criteria – Part B

Inclusion criteria	1. All inclusion criteria detailed in Table 1 (Part A)
	2. Human leukocyte antigen class I and/or class II DSA-positivity
	3. Morphological and immunohistochemical AMR features (Table 2)
Exclusion criteria	1. All exclusion criteria detailed in Table 1 (Part A)
	2. Laboratory tests
	Thrombocytopenia <30 g/l within 2 weeks before enrolment
	Neutrophil count <1 g/l within 2 weeks before enrolment
	4. Peripheral neuropathy ≥ grade 2
	5. Distinct index biopsy results
	T-cell-mediated rejection classified Banff grade > I
	*De novo* or recurrent severe thrombotic microangiopathy
	Polyoma virus nephropathy
	*De novo* or recurrent glomerulonephritis

AMR, antibody-mediated rejection; DSA, donor-specific antibodies.

### Randomization

Patients will be randomized by computer assignment at a ratio of 1:1 for the two treatment groups (bortezomib versus placebo) using an online randomization tool [35]. To avoid unbalances in baseline variables that potentially affect treatment responses, randomization will be stratified according to eGFR (>50 ml/minute/1.73 m^2^ vs. <50 ml/minute/1.73 m^2^) and the presence or absence of T-cell-mediated rejection (no rejection or borderline lesion vs. Banff I rejection).

### Interventions

Study interventions will be carried out in a double-blinded fashion. We have included the option of a rescue unblinding in case of emergencies.

Patients allocated to the intervention group will receive two cycles of bortezomib at 3-month intervals. Each cycle will consist of bortezomib 1.3 mg/m^2^ administered twice weekly on days 1, 4, 8 and 11. Bortezomib will be injected intravenously and all bortezomib-treated patients will receive oral antiviral prophylaxis to prevent the development of viral infection, particularly herpes zoster infection. Valacyclovir (500 mg/day; patients with eGFR <30 ml/minute/1.73 m^2^, 250 mg/day) will be prescribed for 3 weeks after initiation of each bortezomib cycle. Patients allocated to the control group will receive placebo instead of bortezomib (0.9% sodium chloride solution) or valacyclovir (hard gelatine capsules filled with maltodextrine), respectively.

According to our center standard, all included subjects on therapy with a calcineurin inhibitor (tacrolimus or cyclosporine A) or a mammalian target of rapamycin inhibitor (everolimus or rapamycin), without azathioprine or mycophenolate mofetil, will receive mycophenolate mofetil (initially 2 × 500 mg/day; in the absence of gastrointestinal side effects and significant leukopenia or thrombocytopenia, there will be a stepwise increase of dose to 2 × 1,000 mg/day) to avoid underimmunosuppression. Recipients weaned off steroids will receive low-dose prednisolone (initiation with 10 mg/day, tapered to 5 mg/day within 4 weeks).

### Outcome parameters

The primary endpoint will be the course of calculated GFR (slope) over 2 years. As detailed in Table 4, secondary study endpoints include the course of human leukocyte antigen antibody levels, measured GFR and protein excretion, graft and patient survival, occurrence of acute rejection necessitating treatment, and acute and chronic AMR scores in protocol biopsies performed at 24 months.

**Table 4:** Study endpoints

**Primary outcome**	**Slope of eGFR over 24 months**
Secondary outcomes	Graft and patient survival at 24 months
	Measured GFR (Cr-51-EDTA method) at 24 months
	DSA at 6, 12, 18, and 24 months
	Mean fluorescence intensity levels
	Number of human leukocyte antigen specificities
	Urinary protein excretion (protein/creatinine ratio) at 6, 12, 18, and 24 months
	Occurrence of biopsy-proven acute rejection necessitating rejection treatment
	Acute AMR score in a protocol biopsy performed at 24 months
	Chronic AMR score in a protocol biopsy performed 24 months

AMR, antibody-mediated rejection; Cr-51-EDTA, chromium ethylenediamine tetraacetic acid; DSA, donor-specific antibodies; eGFR, estimated glomerular filtration rate; GFR, glomerular filtration rate.

### Sample size

**Figure 3:**
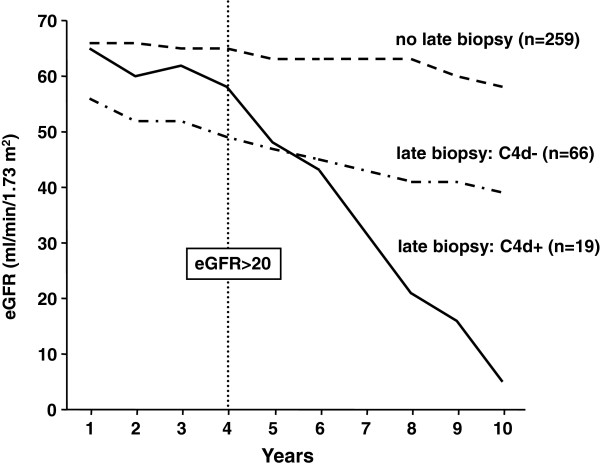
**Course of kidney allograft function in relation to late biopsy results.** In a retrospective cohort analysis, 344 consecutive long-term kidney allograft recipients with a functioning graft at 4 years (transplantation at the Vienna transplant unit) were evaluated for the course of estimated glomerular filtration rate (eGFR). Kidney function is shown in relation to C4 complement split product deposition (C4d) staining results in late indication biopsies performed >6 months after transplantation.

**Figure 4:**
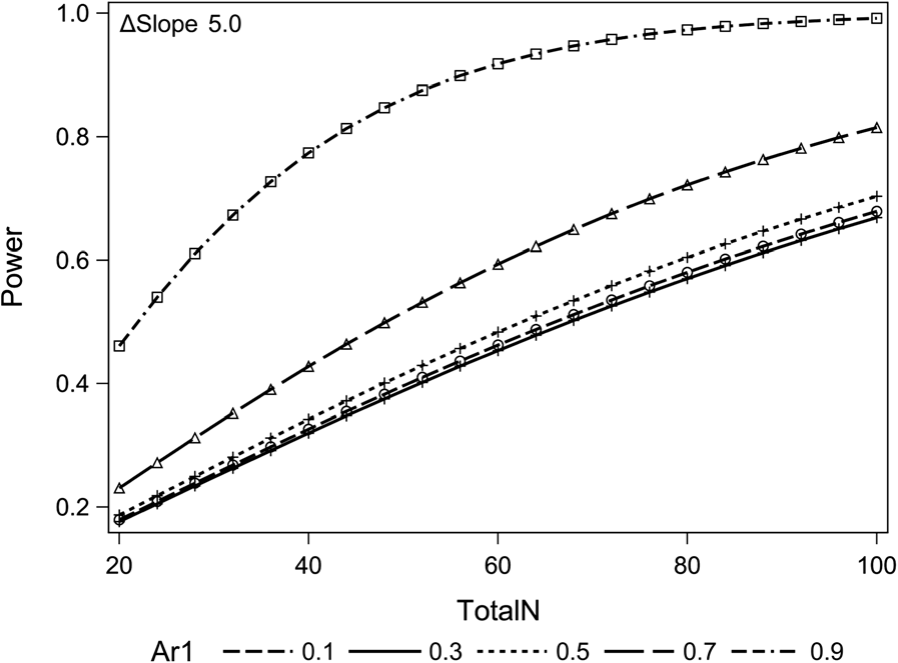
**Sample size determination and power calculation.** Power calculation using five different assumptions of correlation in a covariance matrix. Simulation analyses using 100 permutations of randomly selected 2 × 22 samples of first and second transplants respectively from the Austrian Dialysis and Transplant Registry and the earlier described assumption underline the robustness of our analyses. Seventy-seven of the 100 permutations showed statistically significant delta estimated glomerular filtration rate (eGFR) thresholds of 5 ml/minute/year. Ar1, first-order autoregressive model.

For sample size calculation, we re-analyzed a large retrospective transplant cohort (transplantation and follow-up at the Medical University Vienna; inclusion criteria for analysis: eGFR >20 ml/minute/1.73 m^2^ at 4 years post transplantation, complete follow-up for another 4 years). Evaluating the impact of late (>180 days) C4d-positive AMR on the clinical performance of kidney allografts, we observed a GFR slope of −8.2 ml/year (over a follow-up of 6 years), as compared with a slope of −1.8 ml/minute/year in nonbiopsied and −2.8 ml/minute/year in biopsied C4d-negative subjects (Figure 3). Pilot studies using data from the Austrian Dialysis and Transplant Registry were conducted to estimate the eGFR decline and the variance of sequential eGFR determinations. When using only one eGFR determination at 2 years as the primary endpoint, analyses showed an impracticable high sample size number under the assumption of a median treatment effect of bortezomib (standard deviation 0.5). The difference in slope of half-yearly determined eGFR between the two treatment groups will therefore be used as the quantitative outcomes measure. Mixed linear models for longitudinal data were used for analyses. Power calculation using an autoregressive covariance matrix of the first order with a correlation of 0.9, an alpha value of 0.05 for the interaction term of treatment and time, and an attrition of 8% per year showed that 2 × 22 subjects would be required to uncover a difference in eGFR slopes of 5 ml/minute/year with a power of 80% (Figure 4).

### Data safety monitoring board

This study will be monitored by an independent data and safety monitoring board of the Medical University Vienna. Interim analyses will be performed by the data and safety monitoring board after completion of 10 cases and 20 cases in each treatment group. The Lan and DeMets extension of the O’Brien-Fleming stopping rules will be applied [36] and the trial will be stopped if observed *P* <0.00001 (first interim analysis) or observed *P* <0.00305 (second interim analysis) occurs.

### Quality control and quality assurance

Monitoring procedures, which will include predefined regular visits at the study site, will be carried out by a study monitor. Throughout the study, the investigator will grant access to all source documents including case report forms and other protocol-related documents. Subject confidentiality will be maintained in agreement with local regulations. The monitor will inspect the case report forms at serial intervals following a defined monitoring plan, in order to verify adherence and completeness of the protocol as well as the validity and accuracy of entered data. His responsibility also includes the verification for the presence of informed consent, adherence to the inclusion/exclusion criteria, documentation of severe adverse events and the recording of the main efficacy, safety, and tolerability endpoints. The investigator will resolve discrepancies of data, and upon request he will make all study-related source data and records available to a qualified quality assurance auditor mandated by the sponsor or in the form of competent authority inspectors.

### Safety evaluation and reporting of adverse events

The investigator ensures that adequate medical care is provided in any clinical situation, including emergencies. Safety evaluation includes a careful monitoring of all adverse events, including serious adverse events. As defined by the International Conference on Harmonization guidelines and World Health Organization Good Clinical Practice guidelines, serious adverse events are events that result in patient death, are life-threatening, require or prolong hospital stay, cause persistent or significant disability or incapacity, result in congenital anomaly or birth defect, or necessitate specific interventions.

The most probable and usually transient adverse events caused by bortezomib are mild to moderate thrombocytopenia and leukocytopenia, decreased appetite, gastrointestinal side effects (vomiting, nausea, diarrhea), fatigue, peripheral neuropathy and other neurological symptoms, such as headache, dizziness, or tremor. Intensified immunosuppression may be associated with an increased infection risk (herpes zoster, herpes simplex, pneumonia, bronchitis, sinusitis, nasopharyngitis). A careful patient follow-up will therefore include a complete neurological status awareness of any sign of infection (bacterial, viral and/or fungal).

The most probable adverse events caused by valacyclovir are headache, fever, nausea, gastrointestinal side effects (vomiting, diarrhea, abdominal pain), dizziness, hallucination, confusion, changes in blood cell counts (leukopenia, thrombocytopenia, anemia) and increased liver and/or kidney parameters.

### Methodology

#### Human leukocyte antigen antibody detection

Sera will be prescreened for anti-HLA IgG using LabScreenMixed assays (One Lambda, Canoga Park, CA, USA). For identification of HLA antigen/allele specificities, prescreen-positive samples will be subjected to single-antigen flow-bead testing (LABscreen Single Antigen assays; One Lambda). Test results will be documented as mean fluorescence intensities, and mean fluorescence intensity >1,000 will be considered positive. Donor-specificity will be defined according to donor and recipient HLA typing results.

#### Transplant biopsies

Index and 24-month protocol biopsies will be performed after exclusion of a coagulation disorder or thrombocyte counts below 80%. Anticoagulants or inhibitors of thrombocyte aggregation will be transiently paused in the context of biopsy. The biopsy will be performed under local anesthesia using ultrasound-guided percutaneous techniques (two cores per biopsy, 16 G needle). After biopsy, patients will be monitored closely for at least 6 hours for any complications (serial blood pressure measurements, monitoring for hematuria, control of blood picture 4 hours after biopsy). Biopsies will be evaluated on standard paraffin-embedded sections including immunohistochemical C4d staining. In addition, biopsy samples will be evaluated by electron microscopy.

#### Assessment of kidney function

The eGFR will be calculated using the Mayo equation [37]. For kidney transplants, this equation was reported to be superior with respect to estimations of GFR slopes [38]. The chromium ethylenediamine tetraacetic acid GFR will be assessed by measuring the clearance of chromium-51 ethylenediamine tetraacetic acid according to a standard protocol.

#### Statistical methods

All analyses will be conducted according to the intention-to-treat principle. Continuous data will be analyzed by *t* test, and categorical data by a chi-square test or Fisher’s exact test when appropriate. We will perform stratification for both treatment groups (baseline eGFR and T-cell-mediated rejection, Banff grade I) during analysis of the serum creatinine trajectories using a mixed linear model with time and therapy as the independent parameters. The most applicable covariance matrices will be determined by graphical analysis and evaluated by the log-likelihood ratio. Data missing at random are expected to account for less than 5% of parameters and will be addressed by multiple imputation. Sensitivity analyses will be performed to test the violation of the missing at random assumption. For all analysis, SAS for Windows 9.2 (The SAS Institute Inc., Cary, NC, USA) will be used.

### Ethical issues

The BORTEJECT Study will be conducted in accordance with the principles of the Declaration of Helsinki 2008. Institutional ethics committee approval (EK1515/2012) was obtained for all aspects of the study. All study participants will be asked to sign the written informed consent in order to participate in the study (patient insurance included). We will adhere to all the trial-related requirements, good clinical practice requirements (International Conference on Harmonization Good Clinical Practice), good laboratory practice and the applicable regulatory requirements.

### Study registration

The study was approved by the Austrian regulatory authority (Federal Office for Safety in Health Care, Austrian Agency for Health and Food Safety, AGES reference number: LCM-717840-0001) and was registered in the European Clinical Trials Database (EudraCT number: 2012-002857-41) and in a public clinical trial database (ClinicalTrials.gov NCT01873157).

## Discussion

The prevention and treatment of late AMR represents a major challenge in transplant medicine. In the last decade, ongoing humoral rejection has been recognized as one of the cardinal causes of long-term kidney allograft dysfunction and loss [11-15]. The diagnosis of AMR has been refined by the establishment of clear-cut diagnostic morphologic and immunohistologic criteria [6,7] and the implementation of innovative techniques allowing for the precise detection and characterization of deleterious HLA reactivity patterns [39]. Nevertheless, there is still a need for efficient therapeutic strategies to counteract the development of irreversible graft damage triggered by humoral alloresponses. The establishment of efficient protocols for the treatment of chronic rejection would have a major impact on standard clinical care in organ transplantation and would probably improve graft survival in a considerable proportion of transplant recipients. However, up to now, no systematic trial to prove clinical efficiency of targeted anti-humoral therapy has been reported.

In the last years, uncontrolled studies have suggested a beneficial effect of distinct treatment approaches on the course of late AMR. Very recently, Billing and colleagues reported on a cohort of 20 pediatric kidney transplant recipients who were diagnosed as having C4d-positive chronic AMR [22]. In support of smaller previous series [20,21], combined treatment with high-dose IVIG and CD20 antibody rituximab stabilized kidney function and led to a decrease in DSA levels at least in some of the treated patients. Even though a few recipients lost their graft during follow-up, longitudinal analysis of kidney function showed an amelioration of the decline in GFR, particularly in patients with a lesser degree of chronic injury. Major caveats of this and of other studies, however, are an uncontrolled design and the lack of a nontreated control group, respectively [22].

While IVIG and rituximab as well as other treatments may not target long-lived antibody-producing plasma cells [24], the proteasome inhibitor bortezomib was shown to directly affect the function and integrity of nonmalignant plasma cells [25-27], and may therefore be a promising approach to halt the progression of antibody-mediated graft damage. This agent was shown to directly block HLA antibody production in the context of acute AMR [27], and numerous case reports and uncontrolled studies have pointed to efficiency of this approach in the treatment of acute rejection [28-30]. Such promising preliminary data and a recently published case of chronic AMR showing partial responses to bortezomib treatment, including DSA reduction and loss of C4d staining along transplant capillaries [32], have now prompted us to plan a systematic trial designed to analyze the concept of proteasome inhibition.

Applying a randomized placebo-controlled double-blind design, our single-center trial has the potential to definitely clarify the impact of bortezomib on the course of late AMR after kidney transplantation. A major point is that the trial is investigator initiated and is therefore free from any commercial bias.

Considering earlier reported rates of DSA positivity in long-term transplant recipients [13], the chosen approach of a cross-sectional screening of a large number of patients followed at our outpatient facility can be expected to allow for the recruitment of a sufficient number of patients fulfilling the criteria for randomization in the intervention trial.

To avoid imbalances between study groups, randomization will be stratified for kidney function at the time of randomization and the presence or absence of T-cell-mediated rejection in index biopsies. Stratification for kidney function (GFR <50 ml/minute/1.73 m^2^ vs. >50 ml/minute/1.73 m^2^) was chosen to account for expected interindividual variability regarding the extent of chronic transplant injury (for example, transplant glomerulopathy, chronic vasculopathy or interstitial fibrosis/tubular atrophy). Indeed, one may argue that an irreversibility of chronic injury may critically influence the responsiveness to specific treatment. Similarly, intergroup differences with respect to the proportions of study patients with acute T-cellular rejection could substantially influence trial outcomes.

We have chosen the slope of GFR over 24-month follow-up as the primary endpoint. Using kidney function as a surrogate parameter of long-term allograft survival [40], the sample size calculation revealed the need for inclusion of 44 patients to detect clinically relevant GFR differences between groups (5 ml/minute/1.73 m^2^ per year). We are aware that choosing long-term kidney transplant survival (for example, at 5 years) as a hard primary endpoint may not be practical considering the need for large sample sizes, extensive recruitment periods and excessive asset costs of study medication.

If our study proves that bortezomib can effectively block DSA production and consequently improve transplant outcomes, this finding will markedly challenge current immunosuppressive standards. Considering the high incidence of circulating donor-specific alloreactivity and biopsy-proven AMR in long-term organ transplants, our proof-of-concept study may have the potential to provide a valuable basis for treatment guidelines improving long-term allograft outcomes.

### Trial status

Recruitment of study patients (cross-sectional screening) started on 14 October 2013. We expect completion of enrolment in September 2014.

## Abbreviations

AMR: antibody-mediated rejection; C4d: C4 complement split product deposition; DSA: donor-specific antibodies; eGFR: estimated glomerular filtration rate; GFR: glomerular filtration rate; HLA: human leukocyte antigen; IVIG: intravenous immunoglobulin.

## Competing interests

The authors declare that they have no competing interests.

## Authors’ contributions

FE, GB and GAB were responsible for conception and design, data collection and analysis, financial support, manuscript writing and final approval of the manuscript. ES, ZK and CW were responsible for data collection and analysis, critical revision and final approval of the manuscript. MW and HR were responsible for data collection and analysis, critical revision and final approval of the manuscript. LM, HH and RO were responsible for data analysis, critical revision and final approval of the manuscript. All authors read and approved the final manuscript.
